# EDTA-Induced Pseudothrombocytopenia up to 9 Months after Initial COVID-19 Infection Associated with Persistent Anti-SARS-CoV-2 IgM/IgG Seropositivity

**DOI:** 10.1093/labmed/lmab050

**Published:** 2021-08-20

**Authors:** Dániel Bereczki, Béla Nagy, Adrienne Kerényi, Gábor Nagy, Krisztina Szarka, Katalin Kristóf, Balázs Szalay, Barna Vásárhelyi, Harjit P Bhattoa, János Kappelmayer

**Affiliations:** 1 Department of Neurology, Semmelweis University, Budapest, Hungary; 2 MTA-SE Neuroepidemiological Research Group ELKH, Budapest, Hungary; 3 EANcore COVID-19 Task Force, European Academy of Neurology, Vienna, Austria; 4 Department of Laboratory Medicine, Faculty of Medicine, University of Debrecen, Debrecen, Hungary; 5 Faculty of Pharmacy, University of Debrecen, Debrecen, Hungary; 6 Department of Medical Microbiology, Faculty of Medicine, University of Debrecen, Debrecen, Hungary; 7 Department of Laboratory Medicine, Semmelweis University, Budapest, Hungary

**Keywords:** EDTA-induced pseudothrombocytopenia, SARS-CoV-2 virus infection, COVID-19, persisting anti-SARS-CoV-2 IgM/IgG seropositivity, antinucleocapsid antibody, anti-Spike 1 receptor binding domain (anti-S1-RBD), anti-Spike 1 and Spike 2 (anti-S1/S2) IgG test

## Abstract

Platelets have a role in vascular complications of COVID-19-related viral coagulopathy. Although immune-induced thrombocytopenia has been described mostly in moderate-to-severe COVID-19, the prognostic role of platelet count in COVID-19 is still controversial. Pseudothrombocytopenia has been reported to represent COVID-19-associated coagulopathy in critical illness, and transient EDTA-dependent pseudothrombocytopenia lasting less than 3 weeks was described in a patient with severe acute COVID-19 pneumonia. In our case study, EDTA-induced pseudothrombocytopenia was still present at 9 months after an initial SARS-CoV-2 virus infection in an apparently recovered 60 year old man. The persistence of antinucleocapside and antispike antibodies 9 months after the initial infection suggests that EDTA-induced pseudothrombocytopenia may be related to anti-SARS-CoV-2 IgG or IgM antibodies. We should acknowledge the possibility that pseudothrombocytopenia may also appear in some patients after seroconversion after the launch of large-scale vaccination programs.

The COVID-19 disease caused by the SARS-CoV-2 virus is an ongoing pandemic that began in December 2019, resulting in >142 million identified patients and >3 million fatalities through April 20, 2021.^1^ Endothelial cell activation^2^ and viral coagulopathy have an important role in COVID-19-related complications.^3^ Thrombocytopenia at hospital admission is often reported in patients with more severe cases of illness, suggesting increased platelet consumption.^4^ Immune-induced thrombocytopenia has been described as a complication of COVID-19, appearing mostly in moderate-to-severe illness.^5^ In routine clinical care, platelet count is usually determined by hematologic analyzers using EDTA-anticoagulated blood specimens. In rare instances, EDTA induces time- and temperature-dependent in vitro platelet aggregation, resulting in a misleading underestimation of platelet counts termed as EDTA-induced pseudothrombocytopenia.^6^ This phenomenon often occurs because of a conformational change of platelet surface glycoprotein IIb/IIIa (GPIIb/IIIa) induced by EDTA, which allows natural IgM or IgG autoantibodies to bind to the GPIIb/IIIa receptor, leading to platelet agglutination.^7-9^ EDTA-induced pseudothrombocytopenia is only present in vitro and has no known associated clinical significance.^10^ It can be persistent or transient, sometimes showing alternating periods without the generation of aggregates.^11^

The development of EDTA-induced pseudothrombocytopenia can be complicated by COVID-19 disease. Others have reported transient EDTA-dependent pseudothrombocytopenia in a 59 year old woman with severe COVID-19 pneumonia, lasting from the second to the 17th day of hospital treatment.^12^ The plausible reason is that the SARS-CoV-2–specific antibody has the epitope to bind to the cryptic platelet antigen that causes a cross-reaction.^12^ We here report on an apparently recovered patient in whom pseudothrombocytopenia was still present at 9 months after initial SARS-CoV-2 virus infection.

## Case Report

The patient in this case report was a 60 year old man with a past medical history of rapid eye movement sleep–related obstructive sleep apnea syndrome and hypertension. The patient reported compliance with continuous positive airway pressure equipment for his sleep apnea. He developed fever (up to 38.9°C), generalized weakness, ageusia, and anosmia. A polymerase chain reaction (PCR) analysis of the nasal and throat swab identified SARS-CoV-2 infection (Azureseq 200, Omixon Ltd, Budapest, Hungary). Fever lasted for 7 days; fatigue and insomnia diminished over this time period but persisted for several weeks. Arterial blood oxygen saturation assessed by fingertip pulse oximetry was normal initially but dropped to 85% to 87% between day 5 and day 10 of the infection with a respiration frequency of 30 to 34/min. There was no cough, dyspnea, or chest pain. During the 14-day home quarantine there was no need for hospitalization, and only multivitamins and antipyretics were administered in addition to the regular antihypertensive medication. No corticosteroids or agents with potential antiviral effect were used. At the end of the quarantine, the repeated nasal and throat swab PCR tests on 2 consecutive days for SARS-CoV-2 did not identify the presence of the virus, and several further PCR tests up until the end of December 2020 consistently remained negative.

**Figure 1.**
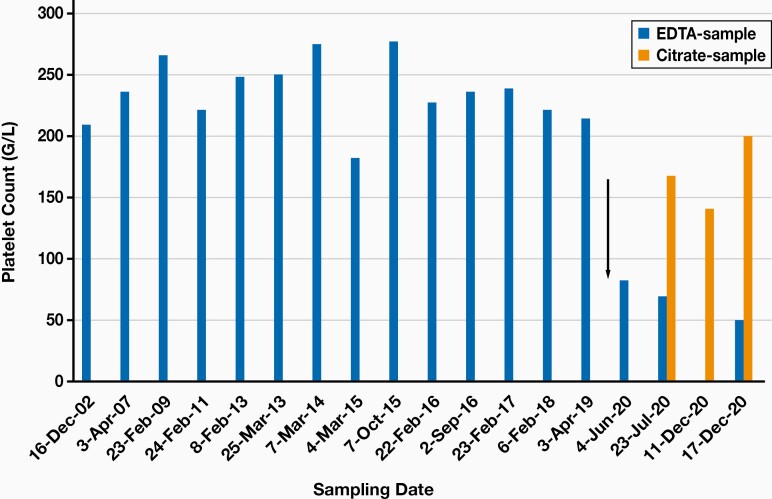
Platelet counts in EDTA- and citrate-anticoagulated specimens in the history of the patient. Arrow marks the time of SARS-CoV-2 infection.

**Figure 2.**
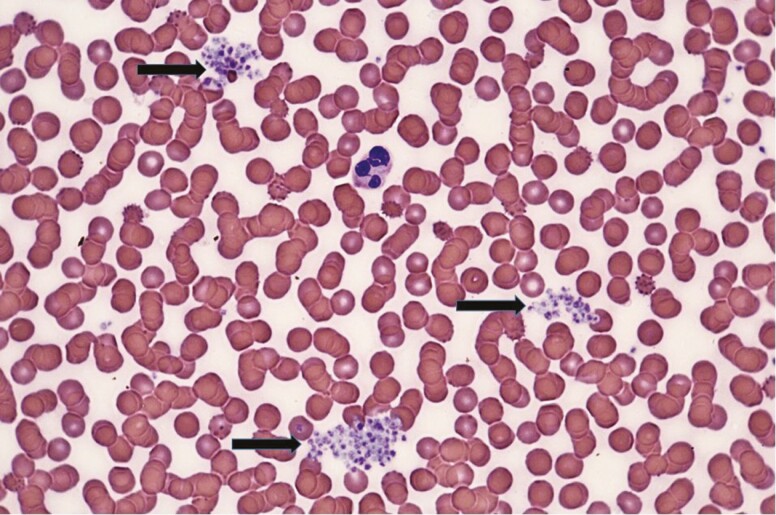
Platelets in the smear of the EDTA-anticoagulated specimen at 9 months after the acute infection. Arrows point to the platelet aggregates. May-Grünwald-Giemsa staining, original magnification.

A laboratory checkup was performed at 2 months after the acute phase, and thrombocytopenia (82 G/L) was detected in the EDTA-anticoagulated whole blood sample on a hematology analyzer (Sysmex 2000 hematology analyzer, Sysmex, Japan). Since 2002, the patient had 14 normal platelet counts evaluated (range, 182–277 G/L); the most recent had been determined 11 months before COVID-19 symptoms (**Figure 1**). At 9 months after the acute SARS-CoV-2 infection, platelet counts were consistently low in EDTA-anticoagulated blood specimens but were practically normal in specimens in which sodium citrate was used for anticoagulation (**Figure 1**). In addition, platelet aggregates were observed in the EDTA-anticoagulated blood smear (**Figure 2**). This phenomenon was established when the repeatedly assessed platelet count increased in citrate-anticoagulated blood (**Figure 1**). From the citrate-anticoagulated blood, the platelet count was calculated by multiplying the platelet result by a factor of 1.1. Pseudothrombocytopenia may not be exclusively an in vitro phenomenon in this patient, because the platelet distribution width increased (69.2 fL; reference range: 25.0–65.0 fL) and the mean platelet volume was in the high-normal range (mean platelet volume = 11.0 fL; reference range: 7.2–11.1 fL).

**Figure 3.**
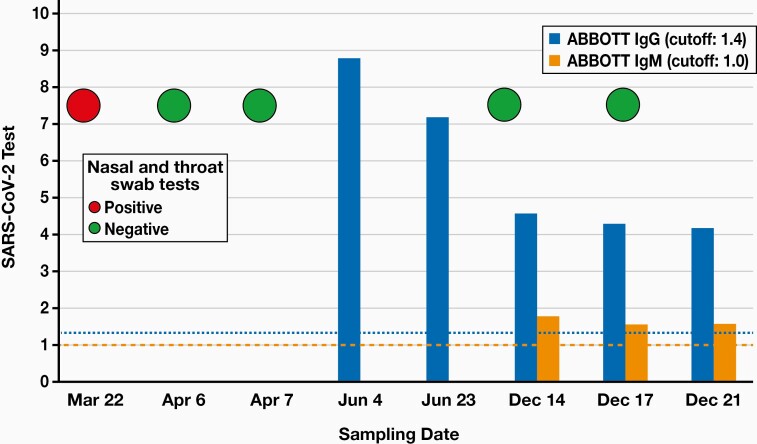
COVID-19 status of the patient with PCR of the nasal-throat swab and serum IgG/IgM status using the Abbott test. PCR, polymerase chain reaction.

Using the Abbott antinucleocapsid SARS-CoV-2 IgG and IgM tests (Abbott Diagnostics, Wiesbaden, Germany), high SARS-CoV-2 IgG titer was detected after 4 months of infection. We then found decreasing levels of anti-SARS-CoV-2 IgG antibody over time (**Figure 3**), but both IgG and IgM antibodies were over the cutoff value even at 9 months after the acute infection. To confirm these results, two different Roche Cobas SARS-CoV-2 serology tests (Elecsys Anti-SARS-CoV-2 and Anti-SARS-CoV-2 S, Roche, Switzerland) were performed in a sample obtained at December 21, 2020 with the following results: anti-nucleocapsid total Ig cut-off index (COI) value: 107.3 at the cutoff value of 1.0, and anti-Spike 1 receptor binding domain (anti-S1-RBD) total Ig titer >250 U/mL at the cutoff value of 0.8 U/mL. In parallel, Diasorin anti-Spike 1 and Spike 2 (anti-S1/S2) IgG test was also used (IgG titer: 91.8 AU/mL, cutoff value: 15 AU/mL) (DiaSorin S.p.A., Saluggia, Italy). All these analyses confirmed the persistence of anti-SARS-CoV-2 antibodies at 9 months after the initial infection.

By 9 months after the acute phase, fatigue and sleep disturbance gradually decreased, and although physical load capacity had not returned to the pre-COVID-19 level, there were no clinical signs of reinfection, and SARS-CoV-2 PCR from whole blood using different methods (automatically using the MagNA Pure 96 DNA and Viral NA Small Volume Kit and manually using the Perkin Elmer SARS-CoV-2 real-time PCR assay, N and ORFlab SARS-CoV-2 targets; and using the Azureseq 200, Omixon Ltd, Budapest, Hungary) in 2 independent laboratories excluded virus persistence in the blood.

## Discussion

The prevalence of EDTA-induced pseudothrombocytopenia is estimated to be 0.03% to 0.27% of the general outpatient population and does not appear to be related to age and sex.^11^ Pseudothrombocytopenia may also be induced by viral infection. As such, hepatitis A virus infection has been the most common culprit, followed by cytomegalovirus and influenza A H1N1 infections.^13^ Transient EDTA-induced pseudothrombocytopenia has also been associated with infectious mononucleosis; the pseudothrombocytopenia phenomenon resolved after 2 months, when the patient recovered from the infection.^14^ Furthermore, pseudothrombocytopenia may be linked to dengue virus infection^15^ or after an acute viral gastroenteritis.^16^ Kuhlman et al^17^ reported a patient with pan-pseudothrombocytopenia and suggested that it may represent COVID-19-associated coagulopathy in COVID-19-caused critical illness. COVID-19 may result in thrombocytopenia via the enhanced activation of platelets, because SARS-CoV-2 infection is associated with an increased surface expression of the GPIIb/IIIa complex of nonstimulated platelets of patients with COVID-19 and the GPIIb/IIIa complex remains upregulated after stimulation.^18^ Consequently, platelet agglutination and clump formation indicate abnormal in vivo platelet activity that increases the risk of microthrombotic disorder.^17^ In contrast, in COVID-19 disease, a SARS-CoV-2-specific antibody can bind to the cryptic platelet antigen, which causes a cross-reaction of the antigen-antibody, leading to EDTA-induced pseudothrombocytopenia.^12^ It has also been speculated that inflammation may be essential to the generation of this phenomenon. Notably, platelet clumping may resolve with the use of citrate and patient recovery.^12^

Although no other viral testing was performed in our patient, he had no clinical signs or symptoms of any other viral illness that may have caused this pseudothrombocytopenia. In another patient with COVID-19, transient pseudothrombocytopenia was detected along with the appearance of anti-SARS-CoV-2 antibodies and disappeared by day 17, when there were no detectable antibodies in the patient anymore.^12^ In the current patient, pseudothrombocytopenia was observed at 9 months after the initial SARS-CoV-2 infection, and anti-SARS-CoV-2 antibodies were still present. Further, although there has already been 1 previous report on the association between COVID-19 and EDTA-induced pseudothrombocytopenia,^12^ this phenomenon may be a coincidence and should not be considered as an evidence of a causal relationship.

Note that these results in the citrated specimens were still lower than pre-COVID-19 values, which could be a late consequence of former SARS-CoV-2 infection. It remains unclear whether in this patient pseudothrombocytopenia was related to antinucleocapsid or antispike antibodies against the SARS-CoV-2 virus because both types of antibodies were present 9 months after the acute infection.

To date, we know of only 1 reported patient with COVID-19-associated EDTA-induced pseudothrombocytopenia.^12^ In addition to the current case report, we have no information regarding further reports on COVID-19-associated, EDTA-induced pseudothrombocytopenia in either hospitalized or unhospitalized patients. As reported in Li et al,^12^ if low platelet counts are found in a patient with COVID-19 by the routine automatic analyzers in EDTA-anticoagulated blood specimens, then repeating the determination of platelet count in a citrate-anticoagulated blood specimen before a therapeutic decision is made to correct thrombocytopenia is suggested. Furthermore, because thrombocytopenia has been reported after SARS-CoV-2 vaccination,^19-21^ health care professionals should consider that pseudothrombocytopenia—although not observed to date—may also appear in some patients after seroconversion after the start of large-scale vaccination programs.

The study was approved by the Scientific and Research Ethics Committee of the University of Debrecen and the Ministry of Human Capacities under registration number 32568-3/2020/EÜIG. The patient gave written consent to publish his case report.

This study was funded by the GINOP-2.3.2-15-2016-00043 project. The project is cofinanced by the European Union and the European Regional Development Fund. BN is a recipient of the Lajos Szodoray Grant and an OTKA Bridging Fund (Faculty of Medicine, University of Debrecen). 

## Abbreviations

GPIIb/IIIa: glycoprotein IIb/IIIa
